# Forecasting and what-if analysis of new positive COVID-19 cases during the first three waves in Italy

**DOI:** 10.1007/s11517-023-02831-0

**Published:** 2023-06-14

**Authors:** Serena De Ruvo, Gianvito Pio, Gennaro Vessio, Vincenzo Volpe

**Affiliations:** 1 Dept. of Computer Science, University of Bari Aldo Moro, Bari, Italy; 2 Big Data Lab, National Interuniversity Consortium for Informatics (CINI), Rome, Italy

**Keywords:** COVID-19, Coronavirus, Time series forecasting, What-if analysis, Machine learning

## Abstract

**Abstract:**

The joint exploitation of data related to epidemiological, mobility, and restriction aspects of COVID-19 with machine learning algorithms can support the development of predictive models that can be used to forecast new positive cases and study the impact of more or less severe restrictions. In this work, we integrate heterogeneous data from several sources and solve a multivariate time series forecasting task, specifically targeting the Italian case at both national and regional levels, during the first three waves of the pandemic. The goal is to build a robust predictive model to predict the number of new cases over a given time horizon so that any restrictive actions can be better planned. In addition, we perform a what-if analysis based on the best-identified predictive models to evaluate the impact of specific restrictions on the trend of positive cases. Our focus on the first three waves is motivated by the fact that it represents a typical emergency scenario (when no stable cure or vaccine is available) that may occur when a new pandemic spreads. Our experimental results prove that exploiting the considered heterogeneous data leads to accurate predictive models, reaching a WAPE of 5.75% at the national level. Furthermore, in the subsequent what-if analysis, we observed that strong all-in-one initiatives, such as total lockdowns, may not be adequate, while more specific and targeted solutions should be adopted. The developed models can help policy and decision-makers better plan intervention strategies and retrospectively analyze the effects of the decisions made at different scales.

**Graphical abstract:**

Joint exploitation of data on epidemiological, mobility, and restriction aspects of COVID-19 with machine learning algorithms to learn predictive models to forecast new positive cases.

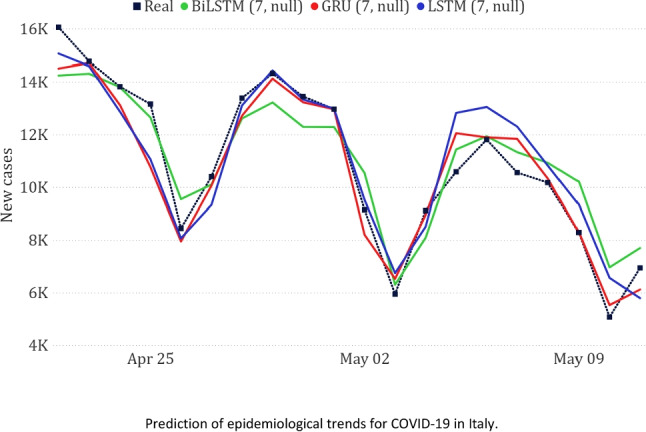

## Introduction

Initially appearing in Wuhan, China, in December 2019, the novel coronavirus has spread rapidly worldwide, leading to an ongoing pandemic known globally as COVID-19.^1^ Typical symptoms, which can appear two to fourteen days after the exposure to the virus, often include fever, cough, fatigue, breathing difficulties, and, in severe cases, they can develop into critical conditions, also leading to death.^2^ Older people have a higher risk of developing severe symptoms among the population. Unfortunately, COVID-19 is easily transmitted when people breathe air contaminated with droplets and small airborne particles containing the virus. The same is true when people touch their eyes or nose after coming into contact with contaminated surfaces.^3^ The rapid spread of the disease has also been exacerbated by the fact that a fraction of people who have been infected show no noticeable symptoms [34], making tracing operations very difficult.

Since the early stages of the pandemic, we have globally observed a severe impact on health infrastructures. This has led policy and decision-makers, in addition to imposing preventive measures such as wearing masks and avoiding gatherings, to declare partial or severe lockdowns in several regions or entire countries. However, long periods of isolation and reduced mobility between cities or regions have also led to dramatic economic and social consequences [32, 41], arousing skepticism about the appropriateness of the adoption of drastic lockdowns as all-in-one solutions [23]. At the time of writing, massive vaccination campaigns have been launched around the world, but the pandemic is still spreading.^4^

In this context, developing predictive models could support policy and decision-makers significantly. Several attempts have been made in this direction using more classical statistical and epidemic models (see Section 2), but they mainly exploit only epidemiological trends (i.e., new positives, number of deaths, etc.). On the other hand, the possible exploitation of additional data related to mobility and applied restrictions has not received the right attention in previous works. This paper proposes adopting machine learning solutions based on multiple heterogeneous data sources to build robust predictive models. Furthermore, we aim to leverage knowledge about the current pandemic by retrospectively analyzing the effects of decisions made, thus supporting the development of more robust strategies to fight against future, although undesirable, outbreaks.

The joint exploitation of data related to epidemiological, mobility, and restriction aspects of COVID-19 with machine learning algorithms is beneficial because it allows us to capture the complex interaction of different factors that influence the spread of the disease. Epidemiological data, such as the number of deaths, provide valuable information about the virus transmission rate and the pandemic’s severity. On the other hand, information about mobility, such as the number of people traveling from one place to another, can help to understand how the disease spreads geographically. Finally, data related to applied restrictions, such as specific measures taken by the government to limit the spread of the virus, can provide insights into the effectiveness of these interventions. Machine learning algorithms can identify patterns and relationships in the data that are difficult to detect with traditional mathematical and statistical methods. In addition, machine learning algorithms can handle the high dimensionality and complexity of the data, which may be essential to model the disease’s spread accurately.

In summary, this paper contributes along the following directions:

- We focus on the Italian case during the first three waves. The country is subdivided into regions with heterogeneous social, economic, and environmental conditions, which have determined significantly different responses and outcomes to the emergency. Although Italy has been the subject of some studies at the national level (e.g., [12, 46]), studies at the regional level are much more limited. We argue territorial specificity, which implies more or less drastic containment measures, cannot be neglected in constructing an accurate predictive model. Therefore, we trained different predictive models at national and regional levels.
- Contrary to previous relevant works mainly focusing on clinical data and mathematical models [7, 29, 47], we integrate data from multiple heterogeneous sources and learn predictive models through machine learning algorithms that also consider the mobility conditions and the imposed restrictions. Considering such relevant factors may significantly support the modeling of this complex phenomenon.
- We show the results achieved by several multivariate time series forecasting models aimed at predicting the amount of new positive cases daily. Note that by relying on a multi-step ahead *recursive* approach [3, 13], the prediction for a given day can be exploited as a training instance to build predictive models for longer-term horizons, e.g., to forecast the epidemiological trend in the following 7 or 14 days.
- Finally, exploiting the most effective learned models, we conduct a what-if analysis to evaluate the impact that different mobility and restriction scenarios could have had on the spread of the virus. Except for some preliminary attempts (e.g., [28, 35, 40]), this type of analysis has not been systematically carried out in the literature, but it can be valuable for retrospectively evaluating the effectiveness of more or less severe decisions.

The rest of this paper is structured as follows. Section 2 reviews existing studies in the literature related to this work. In Sections 3 and 4 we describe the data considered and the experimental setup. Section 5 presents and discusses the obtained results. Finally, Section 6 concludes the paper and outlines the study’s limitations and future developments of this research.

## Related work

Research on the use of Artificial Intelligence (AI) to support decision-making has a long tradition. Supporting decision-makers involves collecting and analyzing evidence, identifying and diagnosing problems, proposing possible courses of action, etc. With AI, these tasks can be done accurately and efficiently by processing huge volumes of data. This is especially important in clinical and biomedical scenarios, where AI can enhance humans’ ability to collect, understand, and make inferences about clinical/biomedical data to try to make optimal decisions. Recently, notable applications of clinical decision support systems have been reported [4, 25, 30, 38]. Building on this experience, since the beginning of the pandemic the research community has made a huge research effort to face the ongoing crisis. Computer vision methods have been applied to computed tomography and radiological imaging to support faster and more reliable diagnosis and prognosis [2, 45]. Natural language processing has been successfully employed for better information retrieval and literature-based discovery [8]. Time series forecasting has been applied to build models capable of predicting epidemiological trends. Our work is framed in the latter context.

A fraction of the literature addressing the problem of developing predictive models for COVID-19 emerged from the very early stages of the pandemic. From this body of knowledge, several works have adopted statistical and epidemic models to help understand and manage the pandemic’s evolution and predict transmission scenarios. For example, [7, 26, 29, 36, 47] applied classic mathematical models to provide quantitative guidance in the application of control measures in China, India, and South Korea. While these models can effectively capture the crucial characteristics of infectious diseases, they are typically based on basic assumptions, such as susceptibility to infection or recovery rate, which may only hold in some scenarios. On the other hand, pure sequential methods designed for time series forecasting, such as the popular Autoregressive Integrated Moving Average (ARIMA), can better uncover hidden patterns from training data. Successful applications on the specific case of COVID-19 include the works [10, 14, 44]. However, in recent years, several supervised machine learning approaches have begun to replace more classical methods for this type of problem [5], even in the specific case of COVID-19 forecasting (e.g., [9, 21, 39]). Popular methods, such as Random Forests and LSTMs, may be even better suited to take advantage of non-linear relationships within multidimensional data and can further increase predictive performance. In this work, we follow this direction.

Unfortunately, while many papers have successfully applied various models to predict the epidemiological trend, fewer attempts have been made in the simultaneous exploitation of multiple heterogeneous data sources, as we do in this paper. Moreover, only a few works performed what-if analyses aimed at studying alternative scenarios to actual measures. Some studies have adopted mathematical simulation models to explore the effects of intervention measures [48], vaccination coverage [18], possible reinfection scenarios [31], or the spread of the virus in universities in different conditions [19]. The works most closely related to ours are [28] and [40]. In [28], several machine learning techniques were applied to US demographic, environmental, and mobility data to assess the impact of mobility on COVID-19 at both the national and county level, although only a short period of two months was considered. In [40], a deep learning model was proposed to assess and predict the impact of various lockdown policies on COVID-19 cases, based on applying a clustering approach on countries (emphasizing those with similar lockdown policies) and then focusing on the case of Qatar. In both studies, the Italian case, with its specific territoriality, was not the study’s objective. In this specific context, Parolini et al. [35] recently proposed a mathematical dashboard for analyzing data for the Italian COVID-19 epidemic, but they did not exploit the potential of machine learning models.

From a methodological point of view, we exploit machine and deep learning algorithms to derive predictive models, as done in [9, 21, 39]. In addition, as reported in [40], we exploit the developed models to perform what-if analyses. Compared to these works, the main difference in this study lies in the heterogeneity of the data considered, which concerns the specific Italian case at national and regional levels. In fact, to the best of our knowledge, this study can be considered the first attempt to exploit (i) epidemiological, (ii) mobility, and (iii) restriction data to learn forecasting models, and to perform subsequent what-if analyses, specifically targeting the first three waves in Italy, both at national and regional levels.

## Materials

This section describes the data we collected to perform the study. Several public datasets have been considered, provided by different authorities, research consortia, and companies, covering multifaceted aspects of the COVID pandemic from 2020/03/01 to 2021/05/11 in Italy. This time interval is long enough to cover the first three waves that hit the country. Specifically, the collected data ranges from *epidemiological* attributes (e.g., number of new cases, number of deaths, number of new hospitalizations) to data representing *mobility* trends and the specific *restrictions* imposed at the national or regional level. It is worth noting that although these datasets represent an invaluable resource for subsequent analyses, their combined exploitation required significant efforts for their integration. Therefore, in the following subsections, we describe the steps we followed to perform data collection, data integration, and data validation, emphasizing the differences in terms of type of data, peculiarities, and identified issues. An overview of the features considered is given in Table 1.

### Data collection

#### Epidemiological data

Epidemiological data were collected from two different sources: the dataset provided by the Italian Civil Protection^5^ and the dataset made available by Our World in Data.^6^ The former was curated by the Italian Ministry of Health and contains data on the national, regional, and provincial epidemiological trends, updated daily. In this study, as previously mentioned, we focused on national and regional data. Since we were interested in studying the daily trends, we transformed the three cumulative features shown in Table 1 (i.e., recovered patients, total deaths, and total tests) into non-cumulative daily features.

**Table 1:** Features considered in this study

**Feature**	**Description**	**Source**	**National**	**Regional**	**Type**
Symptomatic patients	Number of symptomatic patients	ICP	✓	✓	D
ICU patients	Number of ICU patients	ICP	✓	✓	D
Hospitalized patients	Number of hospitalized patients	ICP	✓	✓	D
Home isolation	Number of people in home isolation	ICP	✓	✓	D
Total cases	Number of subjects currently positive	ICP	✓	✓	D
Total cases variation	Variation of new cases compared to the previous day	ICP	✓	✓	D
Recovered patients	Total number of recovered patients	ICP	✓	✓	D
Total deaths	Total number of deaths	ICP	✓	✓	D
Total tests	Total number of tests performed	ICP	✓	✓	D
New cases	Number of new positive cases (target variable)	ICP	✓	✓	D
Rt	Reproduction rate	OWD	✓	✗	C
Retail and recreation	Travel trends for restaurants, bars, etc	GCM	✓	✓	D
Grocery and pharmacy	Travel trends for grocery and food stores, etc	GCM	✓	✓	D
Parks	Travel trends for parks, gardens, etc	GCM	✓	✓	D
Transit stations	Travel trends related to hubs of public transport	GCM	✓	✓	D
Workplaces	Travel trends related to workplaces	GCM	✓	✓	D
Residential	Time spent in residential places	GCM	✓	✓	D
Driving	% of requests for driving directions	AMT	✓	✓	D
Walking	% of requests for walking directions	AMT	✓	✓	D
Transit	% of requests for public transport directions	AMT	✓	✓	D
School closing	Closure of schools and universities	OCG	✓	✗	O
Workplace closing	Closure of workplaces	OCG	✓	✗	O
Public event cancellation	Cancellation of public events	OCG	✓	✗	O
Restrictions on gatherings	Restrictions on gatherings	OCG	✓	✗	O
Public transport closing	Closure of public transport	OCG	✓	✗	O
Stay at home	Obligation to stay at home	OCG	✓	✗	O
Restrictions on internal movement	Travel restrictions between cities and regions	OCG	✓	✗	O
International travel controls	Restrictions on international travel to the country	OCG	✓	✗	O
Testing policy	Policies on who has access to tampons	OCG	✓	✗	O
Vaccination policy	Policies for the administration of vaccines	OCG	✓	✗	O
Government response index	Degree of government response to contain infections	OCG	✓	✗	C
Color zone	Indicator of containment measures	–	✗	✓	O

For each of them, we report a description, its source (ICP = Italian Civil Protection, OWD = Our World in Data, GCM = Google COVID-19 Mobility, AMT = Apple Mobility Trends, OCG = Oxford COVID-19 GRT), the available granularity (if national or regional), and the type (O = ordinal, D = discrete, C = continuous). It is worth noting that features from OCG are ordinal from lowest (no restrictions) to highest (severe restrictions), except for the *government response index*, which represents a summary of all other indicators

**Fig. 1:**
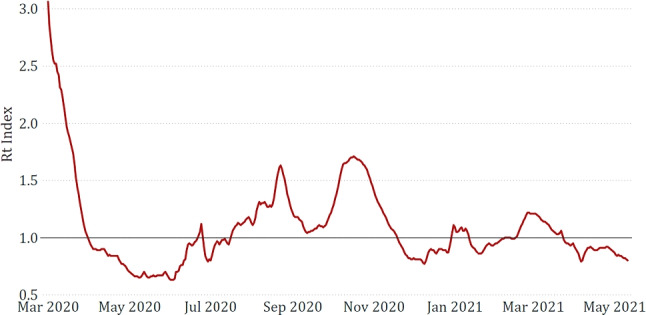
*Reproduction rate* in Italy from March 2020 to May 2021

The dataset provided by Our World in Data contains several epidemiological features related to continents and countries, updated daily. From this dataset, we only selected the *reproduction rate*, which represents an estimation of the Rt index, carried out with the method proposed in [1]. Features representing other relevant information are already collected in the Italian Civil Protection dataset, which also offers these data at a regional granularity level. A graphical representation of the trend of the national reproduction rate in the considered period is shown in Fig. 1.

#### Mobility data

Data about mobility trends were collected from the Google COVID-19 Mobility^7^ and Apple Mobility Trends^8^ datasets. Google Maps uses aggregated and anonymous data to analyze the average number of people in specific categories of places per hour. Such information can play a vital role in making essential decisions against outbreaks. Data from the Google COVID-19 Mobility dataset are provided at a national, regional, and provincial granularity but are also available globally. The data offered by this dataset were updated every 2–3 days, as they were subject to some preprocessing steps before being offered to the public. All the features listed in Table 1 represent the shift, in terms of visitors to specific locations, from a baseline representing the pre-pandemic period. It is worth noting that the information is missing for some days of the year and in some regions due to the lack of sufficient data to aggregate.

Analogously, the dataset offered by Apple was generated by counting the number of requests and driving directions on Apple Maps in specific geographic areas. From a spatial viewpoint, the information is offered at a national, regional, and city level. In our case, we considered all the available features at a national and regional granularity.

#### Restriction data

Mobility trends and the overall spread of the virus have been influenced by restrictive measures put in place by national and regional authorities. Therefore, we also considered data related to the restrictions applied in Italy over time. To this aim, we exploited data offered by the Oxford COVID-19 Government Response Tracker [20]. It collected systematic information on government measures in different countries, including Italy. Several indicators cover different areas in which anti-COVID measures have been adopted, including restrictive and containment measures. The specific indicators used in our analysis are reported in Table 1.

**Fig. 2:**
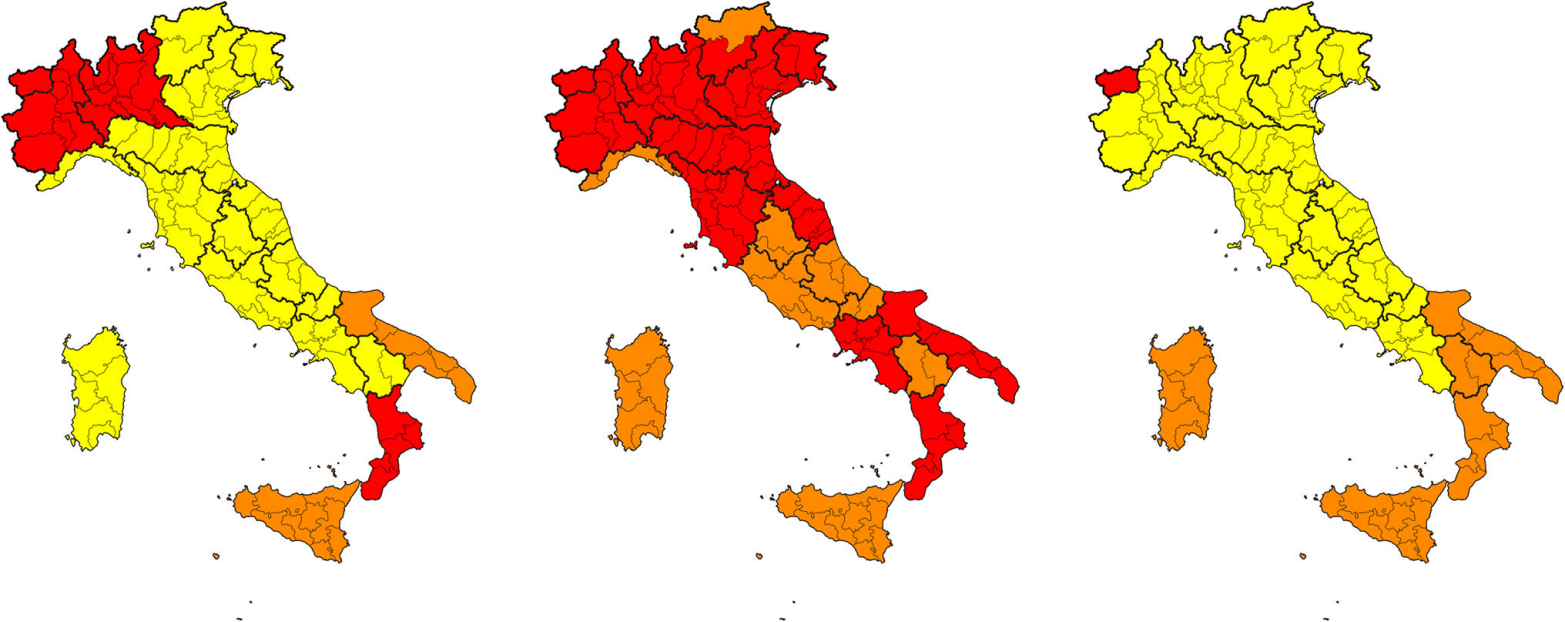
Color zone restrictions imposed on 2020-11-05, 2021-03-29 and 2021-05-06, respectively from left to right

To consider this kind of information also at a regional granularity, an additional dataset was manually created. It contains, for each day, the color zones corresponding to specific restrictive measures established by the Italian Ministerial Decree of 3rd November 2020. The Italian government launched this initiative to take into account the specificity of each region, thus avoiding lockdown at the national level. The color can be one of the following, depending on the severity of the restrictions applied: *white*, *yellow*, *orange*, and *red*. For example, *white* indicated basic restrictions, such as the mandatory wearing of masks, physical distancing, and closure of museums on weekends. At the other end of the spectrum, *red* indicated severe restrictions, including night-time curfews, bans on going out without justification, closure of stores and malls, and distance education. To guarantee consistency from a temporal viewpoint with data related to other perspectives, the dataset was extended to include information about restrictions for dates preceding the Ministerial Decree based on the similarity of the applied restrictions. In particular, from 1st to 4th March 2020, all regions were in the white zone except for Lombardy (orange), Veneto (yellow), and Emilia-Romagna (yellow). From 5th to 9th March 2020, all regions in the white zone switched to the yellow zone. From 10th to 21st March 2020, all regions were assigned to the orange zone, while for the entire lockdown period (from 22nd March to 3rd May 2020), they were assigned to the red zone. From 4th to 17th May 2020, all regions were assigned the orange zone due to the relaxation of restrictions throughout the national territory. From 18th May to 14th June 2020, all regions were assigned to the yellow zone. Finally, for the whole summer and until 21st October 2020, all the regions were assigned to the white zone. Figure 2 shows some examples of the color zones imposed on three different days.

### Data integration

The datasets considered are inherently heterogeneous and require integration steps before they can be properly analyzed. We started with converting names and formatting dates uniformly. In addition, to handle consistent and temporally aligned data, 1st March 2020 was chosen as the starting point, given that, at the beginning of March 2020, all Italian regions recorded the first cases of COVID-19 (while, at the national level, the threshold of 550 cases per day was already exceeded).

At the end of the integration process, one national dataset and 21 regional datasets (considering the Autonomous Province of Trento and Bolzano separately) were obtained. Each row contains data from a single day in both datasets.

### Data validation

**Fig. 3:**
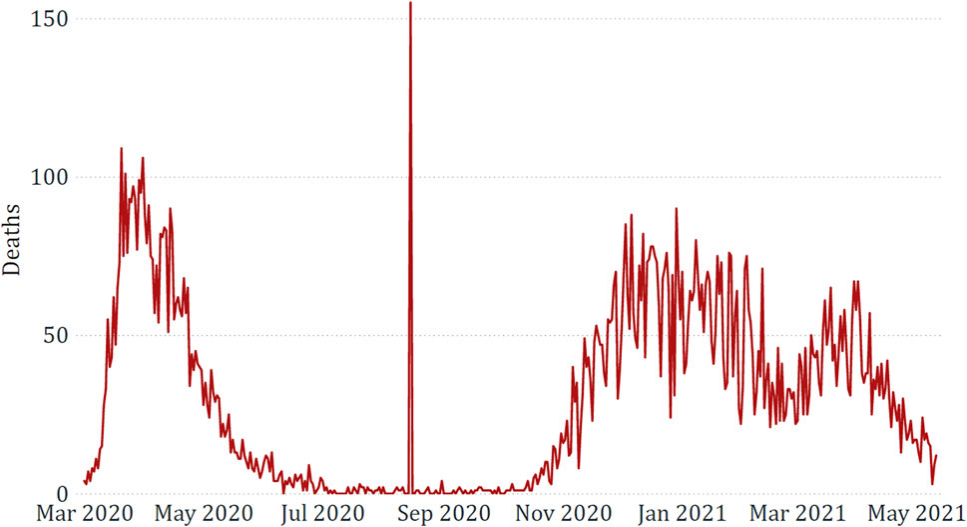
Anomaly found in the epidemiological data related to Emilia-Romagna

**Fig. 4:**
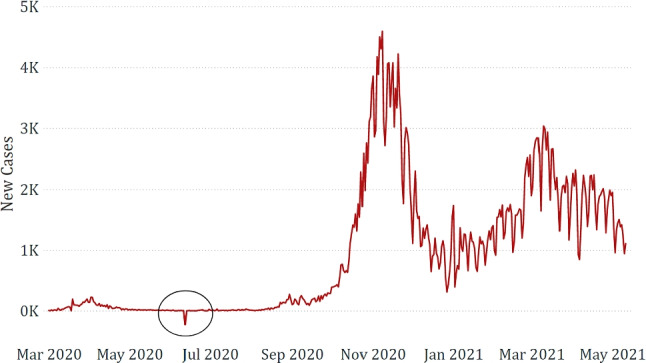
Anomaly found in the epidemiological data related to Campania

The datasets obtained through the integration phase were verified to detect possible errors, outliers, or anomalies. Some anomalies were found in epidemiological data, arguably due to data collection or management issues by the authorities. Some relevant examples are shown in Figs. 3 and 4. We can observe the case of the Emilia-Romagna region, which declared a very anomalous number of new deaths compared to the overall trend, and the case of the Campania region, which declared a negative number of new COVID-19 cases.

As for the other data sources, no critical problems were identified except for sporadic missing periods for some mobility features in some regions. In particular, for the Google Mobility indexes, we found some regions, such as Abruzzo, Molise, Basilicata, Valle d’Aosta, and Umbria, where periods of about 20 consecutive days with missing values for some locations (e.g., parks and transit stations) are present. Moreover, we identified three specific days with no data from Apple Mobility. Unfortunately, there are also regions where these features are completely absent and therefore have not been included in our analysis. The feature *walking* is missing from the Apple dataset for Basilicata, Molise, Valle d’Aosta, Bolzano, and Trento. The feature *driving* is also missing for the latter two regions. In general, we treated anomalies and missing values in two ways: by setting them to a *null* value or by *imputing* them through polynomial interpolation. Note that we treated all features equally and applied these two alternative strategies for handling all the missing values, considering them mutually exclusive.

**Fig. 5:**
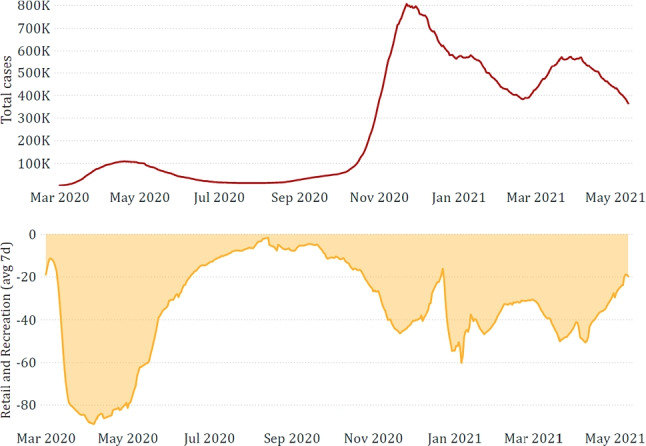
Comparison between *total cases* and *retail and recreation*

**Fig. 6:**
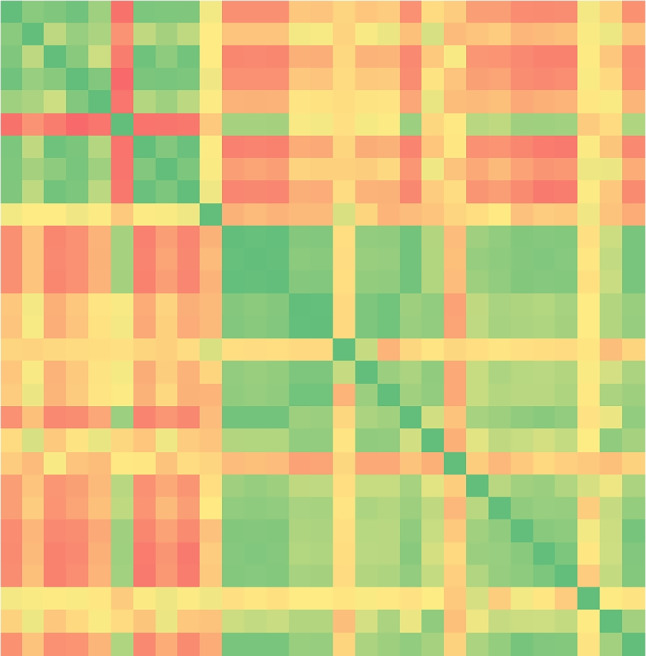
Correlation matrix between features in the national dataset (for better visualization, we show only the corresponding heat map). The color palette ranges from negative correlation (red) to positive correlation (green)

Finally, we performed an exploratory data analysis, which confirmed that epidemiological, mobility, and restriction data are interrelated, thus strengthening our motivation for their combined exploitation. A relevant example is depicted in Fig. 5, which shows how the total positive cases are negatively correlated with retail and recreation as an effect of the restrictions applied. Positive and negative correlations between features can also be observed in the correlation matrix shown in Fig. 6. For the sake of brevity, this figure only depicts national data, but regional data behave analogously. Although some features appear to be correlated, we preferred not to further reduce the available features through feature selection approaches but to let the machine learning algorithms fully exploit all available data.

**Fig. 7:**
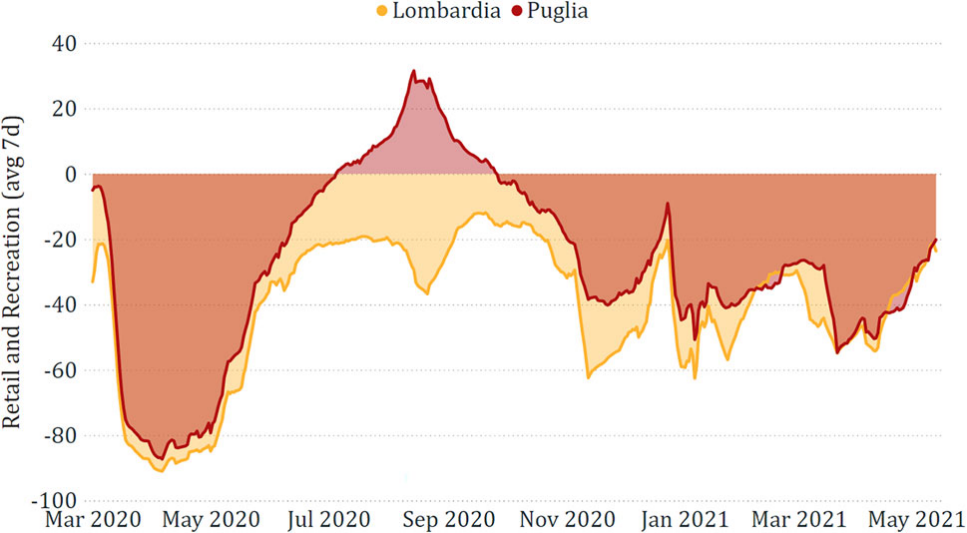
Comparison between *retail and recreation* in Puglia and in Lombardia

The exploratory analysis also confirmed that each region exhibits its peculiarities. In this respect, in Fig. 7, we show how retail and recreation were much higher in Puglia than in Lombardia during the summer of 2020, but this could be due to the fact that the former is a typical tourist region, rather than to aspects related to the pandemic.

**Fig. 8:**
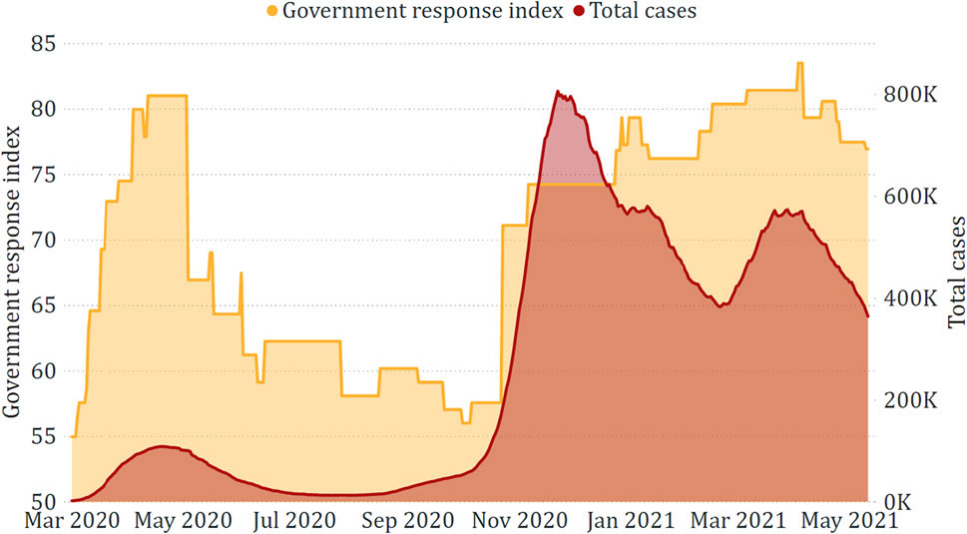
Comparison between *total cases* and *government response index*

Finally, Fig. 8 confirms that the government response to the pandemic has not always been uniform according to the epidemiological trend. After the first strict lockdown, imposed not to saturate the Intensive Care Units, the same severity was no longer applied, although the new positive cases were consistently higher. Indeed, since the autumn of 2020, more specific region-by-region restrictions have been applied.

## Methods

In the following, we describe the approach we followed to achieve the first objective of this work, namely the construction of forecasting models to predict new positive cases. Then, we exploited the resulting models to perform a what-if analysis.

### Learning of forecasting models

Given the features related to epidemiological, mobility, and restriction aspects described in the previous section, our goal has been to learn forecasting models able to predict new positive cases on a day-to-day basis. Specifically, from a machine learning viewpoint, the task considered falls in the category of *multivariate one-step forecasting*. This task can generally be formulated as a regression problem in which the goal is to predict $y_{t+1} \in \mathbb {R}$, at time $t+1$, given a *d*-dimensional feature vector $\textbf{x}_{t} \in \mathbb {R}^{d}$, which also includes the value of the target variable at time *t*. This strategy, commonly known as the *sliding window* method [15], requires defining the size of the window, which plays a crucial role as it determines the contribution provided by old observations and can have a significant impact on the final results. Since, in our specific context, symptoms can arise two to fourteen days after exposure to the virus, corresponding to the time frame required to evaluate the effectiveness of possible restrictions, we learned our forecasting models with a window size $w = 7$ and $w = 14$.

For planning purposes, focusing the task on predicting new cases for the next day can be quite limiting. However, as already introduced in Section 1, a one-step forecasting task can be easily extended to a multi-step forecasting task by adopting a *recursive* approach. Specifically, the predicted value at time *t* can be adopted as a training instance to learn a predictive model for time $t+1$, $t+2$, ..., $t+p$, up to the desired time horizon *p*. Of course, this strategy may lead to the propagation of errors, but it can effectively be adopted to achieve longer-term predictions.

As learning algorithms, we considered the following popular supervised methods:

- *K-Nearest Neighbors* (K-NN) [22], which is a classic instance-based method that does not build a predictive model but computes the target value to assign to a new instance based on the target values of its *k* nearest neighbors in the training set, where the similarity with them is estimated according to the descriptive variables.
- *Support Vector Regression* (SVR), which is a well-known supervised algorithm suitable for both linear and non-linear regression [43]. The name derives from the so-called support vectors, which are training examples selected from the training set that allow identifying an optimal separating hyperplane by solving a quadratic programming problem. When a linear hyperplane may not lead to a good separation, the so-called kernel trick can be adopted, which is based on the computation of a non-linear combination of the original features and the projection of the training examples from the original space into a higher dimensional space via a suitable mapping function.
- *Random Forest* (RF), which is an ensemble method based on the construction of multiple regression trees, each learned from a random sample of training examples and features [6]. Each leaf of a regression tree is associated with a numerical value, representing the prediction provided to new examples falling in such a leaf. On the other hand, internal nodes represent logical conditions defined on the descriptive attributes. The ensemble’s goal is to combine the predictions provided by several “weak” learners to achieve a better generalization capability of the final model.
- *Adaptive Boosting* (AdaBoost), which is an ensemble method that, unlike RF, is based on “boosting” [16]. The goal is to train weak learners sequentially rather than in parallel to obtain a more robust model. More specifically, each learner is trained by focusing on instances not predicted correctly by its predecessor. Therefore, each instance is associated with a weight, which is iteratively adapted, indicating the importance that the next learner should provide.
- *Gradient Tree Boosting*, which is a more recent boosting algorithm that, instead of adapting the instance weights at each iteration as done by AdaBoost, iteratively attempts to fit the new model based on the gradient of a specific loss function, computed on the predictions performed by the previous learner [17]. The adopted loss function must be differentiable and appropriate to the objective to be pursued, such as the Mean Squared Error in the case of regression tasks.
- *Long Short Term Memory* (LSTM) network, which is a recurrent neural network (RNN) architecture suitable for sequential data and specifically designed to model long-term dependencies in the input space through a cell state in addition to the classic hidden state of vanilla RNNs [24]. In LSTMs, information can be explicitly retained or removed from the cell state so that the cell’s internal state remains unchanged if there is no information to retain. Three gates regulate this mechanism: the forget gate, the input gate, and the output gate. LSTMs can be unidirectional or bidirectional (BiLSTM): in the first case, only past information is used; in the second case, the network architecture allows information propagation in both directions.
- *Gated Recurrent Unit* (GRU) network, which is an RNN variant introduced as an improvement on the classic LSTMs [11]. A GRU generally performs similarly or better in terms of efficiency than a normal LSTM, as it reduces the number of operations to be performed. In fact, GRU combines the previous input and forget gate into a single “update” gate.
- *Naive regressor*, which is a simple baseline that naively predicts the number of new cases of the next day as those of the previous day.

While the tree-based methods accept both categorical and numerical data as input, all other methods require additional preprocessing to encode categorical variables to numerical. In this study, the only categorical feature was the color zone in the regional datasets, which, as indicated in Table 1, has been transformed into an ordinal variable with the following encoding: 0 for white, 1 for yellow, 2 for orange, and 3 for red.

**Fig. 9:**
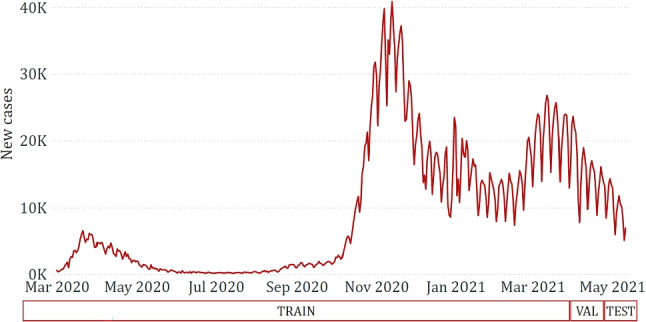
Graphical representation of the applied training-validation-test splitting

Time series forecasting tasks require attention when defining the experimental setting. Indeed, standard evaluation strategies based on cross-validation cannot be directly applied since they could lead to testing splits falling in past periods with respect to training splits. For this reason, as depicted in Fig. 9, we split the available data into:

- Training set: from 2020/03/01 to 2021/04/01;
- Validation set: from 2021/04/02 to 2021/04/21;
- Test set: from 2021/04/22 to 2021/05/11.

Note that, as stated earlier, the test set represents a time horizon that is wider than one single day and, specifically, spans over 20 days. We never considered the real value of any day in the test set while learning the predictive models. On the contrary, following the recursive approach, we adopted the predicted values to learn predictive models for each day of the test set from 2021/04/23 to 2021/05/11.

The adopted split is motivated by the need to exploit as much data as possible for the training phase to learn accurate models based on epidemiological trends observed along multiple waves. The validation set was specifically used to optimize the models’ hyperparameters, thus mitigating the possible occurrence of overfitting issues. Finally, the best-identified hyperparameters were adopted to retrain the models from the combined training and validation sets.

The optimization of the hyperparameters was performed as follows. We applied a grid search that exhaustively explores the hyperparameter space over the validation set for K-NN, SVR, RF, AdaBoost, and Gradient Tree Boosting. On the other hand, we adopted a Bayesian search for methods based on neural networks (i.e., LSTM, BiLSTM, and GRU). The main difference between the Bayesian and classic grid search is that the former focuses on the most “relevant” part of the search space, discarding ranges that are unlikely to provide the best results. For K-NN, the only optimized hyperparameter was the number of neighbors, with values ranging from 1 to 5. For SVR, the search space included the polynomial kernel degree, with values ranging from 1 to 9, and the regularization parameter *C*, which took values of 1, 10, 20, up to 100. For the tree-based methods, we considered the maximum depth of trees, with values ranging from 5 to 15, and the number of trees in the ensemble, with values of 50, 100, 150, and 200. We also looked for the minimum number of samples needed to be in a leaf node and the minimum number of samples needed to split an internal node, with four combinations of considered values, namely (2, 4), (3, 6), (4, 8) and (5, 10). For Gradient Tree Boosting, we experimented with two values for the learning rate, namely, 0.1 and 0.01. Finally, for LSTM, BiLSTM, and GRU, the batch size was set between 16 and 32. The search space also included: the number of epochs, with values ranging from 10 to 100, in steps of 10; the number of hidden units, with values ranging from 8 to 128 in increments of 8; the activation function, set to tanh or ReLU; the dropout rate, with values of 0 and 0.1; the learning rate, which took values between 0.1 and 0.0001. Moreover, we used the Adam optimizer, and the early stopping patience was set to 3 for all neural network models.

To evaluate and compare the obtained performance, we used the following well-known evaluation metrics:

- *Mean Absolute Error* (MAE), which measures the expected value of the absolute error, calculated as $\text {MAE} = \frac{1}{N} \sum _{i=1}^{N} \left| y_{i} - \hat{y}_{i} \right|$, where *N* is the number of samples in the testing set, while $y_{i}$ and $\hat{y}_{i}$ are the true and the predicted values for the *i*-th sample, respectively.
- *Root Mean Squared Error* (RMSE), which measures the square root of the mean of the squares of the errors performed on the testing set; it is computed as $\text {RMSE} = \sqrt{\frac{1}{N}\sum _{i=1}^{N} \left( y_{i} - \hat{y}_{i} \right) ^{2}}$, and generally provides a higher penalization to larger errors with respect to MAE.
- *Weighted Average Percentage Error* (WAPE), which measures the mean absolute percentage deviation of the predicted values from the true values, computed as $\text {WAPE} = 100 \cdot \frac{\sum _{i=1}^{N} \left| y_{i} - \hat{y}_{i} \right| }{\sum _{i=1}^{N} \left| y_{i} \right| }$.

### What-if analysis

Based on the results obtained on the multivariate time series forecasting task, we performed a set of *what-if* analyses to assess the impact of possible different mobility and restriction scenarios on the spread of the virus. We carried out these analyses based only on the best forecasting models, i.e., those that turned out to be the most accurate at testing time, both on the national and the regional data. The adoption of inaccurate models, in fact, would naturally have led to strong distortions in the conclusions. Specifically, two alternative scenarios were investigated, whose specific perturbations are summarized in Table 2:

- *Lockdown*, simulated by perturbing the mobility and restriction features of the test set to mimic the values shown during the first wave of March-April 2020, which, in Italy, led to a total lockdown. It is worth noting that the Italian government has no longer adopted the restrictions applied during this period; instead, specific restrictions have been introduced for each region, as previously mentioned.
- *Reopening*, simulated by perturbing the same features to mimic the values observed during the reopening phase in the summer of 2020 following the lockdown mentioned above.

More precisely, we modified the values of the mobility and restriction-related features of the test set to simulate a different scenario from what actually happened, i.e., to mimic the increase/decrease observed in previous periods of total closure or reopening. Since the time interval of the test set was characterized by a gradual relaxation of restrictive measures in Italy, to simulate a lockdown, it was necessary to decrease the value of mobility-related features and increase that of restriction-related features. Conversely, we increased the value of mobility-related features and decreased that of restriction-related features to simulate a total reopening.

These scenarios were adopted to evaluate how more or less severe restrictions lead to an increase or decrease in new positive cases. In this way, by comparing the new positive cases predicted on the perturbed test set with the actually measured positive cases, we assessed the possible influence of the simulated scenarios.

**Table 2:** Summary of the perturbation introduced in the considered what-if scenarios

**Perturbed feature**	**Lockdown**	**Reopening**
Retail and recreation	$\downarrow$	$\uparrow$
Parks	$\downarrow$	$\uparrow$
Transit stations	$\downarrow$	$\uparrow$
Residential	$\uparrow$	$\downarrow$
Driving	$\downarrow$	$\uparrow$
Walking	$\downarrow$	$\uparrow$
Transit	$\downarrow$	$\uparrow$
School closing	$\uparrow$	$\downarrow$
Workplace closing	$\uparrow$	$\downarrow$
Public transport closing	$\uparrow$	$\downarrow$
Stay at home	$\uparrow$	$\downarrow$
Restrictions on gatherings	$\uparrow$	$\downarrow$
Restrictions on internal movement	$\uparrow$	$\downarrow$

The $\downarrow$ symbol indicates that the feature value has been decreased, while $\uparrow$ indicates that the feature value has been increased. The decrease/increase was estimated on the actual relative changes observed in March-April 2020 and the summer of 2020, respectively

## Results and discussion

In this section, we discuss the obtained results in detail. Specifically, we first show and discuss the results of the multivariate time series forecasting task. Then, we show the outcome of the what-if analysis.

### Results of the forecasting task

We report the results obtained for all dimensions of analysis considered in a public repository (see “Availability”). For the sake of brevity, in the following, we focus our attention on the results of the three best models on the national dataset and on the regions where we achieved interesting predictive performances, i.e., Puglia, Toscana, and Lazio. We specify whether a window size of 7 or 14 days was adopted and whether the missing values and outliers were set to a *null* value or replaced by *imputation*. For each experiment, metrics were calculated on the test set using the best hyperparameter configuration identified on the validation set.

The best-performing models were selected not only based on the quantitative evaluation measures but also according to qualitative comparisons between actual and predicted trends. Indeed, a model may exhibit low error in time series forecasting while largely overestimating or underestimating the underlying trend.

#### National data

**Fig. 10:**
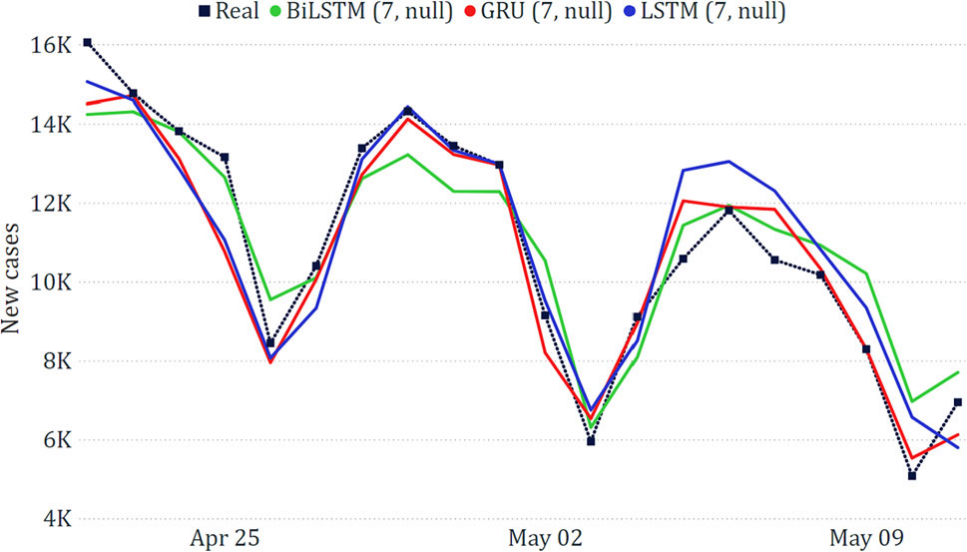
Comparison between actual and predicted epidemiological trends for the national data

In Table 3, we show the results of the three best-performing models on the national dataset. Generally, we can observe that the models based on neural networks led to the best results, with GRU achieving a very low WAPE ($\sim 5\%$). The good obtained results are confirmed by a visual inspection of the predicted trends compared to the real one (Fig. 10).

**Table 3:** Best results on the national dataset

**Model**	**MAE**	**RMSE**	**WAPE**
GRU (7, null)	628.78	879.54	5.75%
LSTM (7, null)	876.47	1084.26	8.02%
BiLSTM (7, null)	887.27	1036.42	8.12%

#### Puglia

**Fig. 11:**
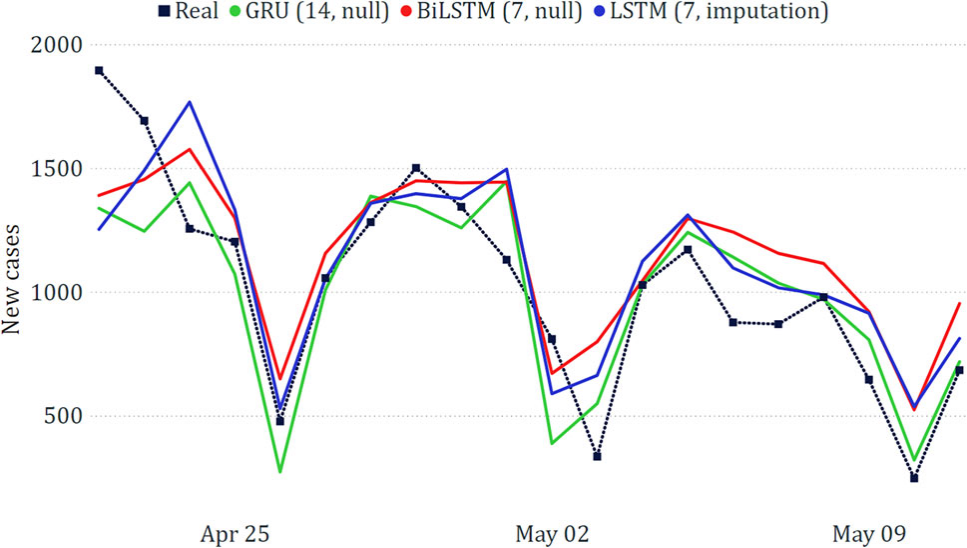
Comparison between actual and predicted epidemiological trends for the Puglia region

The best results obtained for the Puglia region, shown in Table 4 and Fig. 11, confirm the superiority of models based on neural networks. The predicted trends overlap with the real ones, although not perfectly. The errors made by the models are sometimes in a different order of magnitude than those made at the national level; however, it must be considered that the scale of the target variable is much lower (the new positives in the individual regions are much lower than those recorded at the national level). Similar considerations apply to all other regions.

**Table 4:** Best results on the data related to Puglia

**Model**	**MAE**	**RMSE**	**WAPE**
GRU (14, null)	182.64	235.34	17.83%
LSTM (7, imputation)	198.29	256.27	19.36%
BiLSTM (7, null)	216.22	253.70	21.11%

#### Toscana

**Fig. 12:**
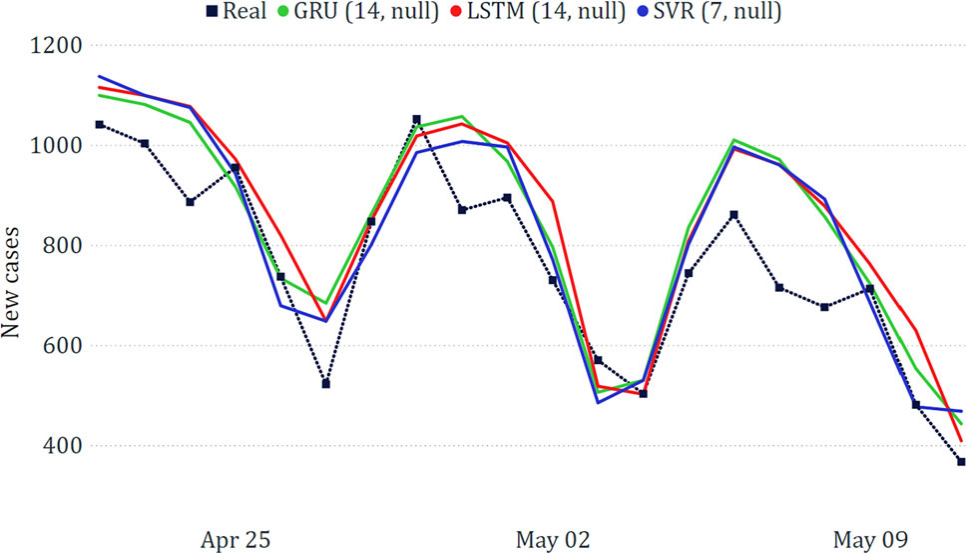
Comparison between actual and predicted epidemiological trends for the Toscana region

Table 5 confirms the predominance of RNNs, although the runner-up is SVR. All models exhibited interesting performances, even though, as shown in Fig. 12, they overestimated the number of new cases towards the end of the testing period.

**Table 5:** Best results on the data related to Toscana

**Model**	**MAE**	**RMSE**	**WAPE**
GRU (14, null)	88.98	112.28	11.73%
SVR (7, null)	93.36	113.60	12.31%
LSTM (14, null)	99.81	120.96	13.16%

#### Lazio

**Fig. 13:**
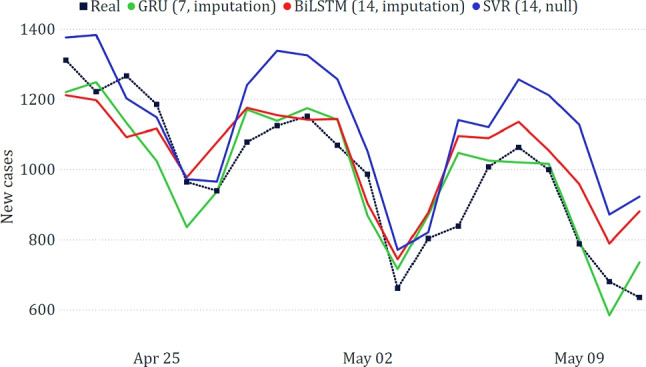
Comparison between actual and predicted epidemiological trends for the Lazio region

For the case of Lazio, GRU was once again the best-performing model (Table 6). We can also notice that SVR exhibited lower performance, largely overestimating the real trend (see Fig. 13).

**Table 6:** Best results on the data related to Lazio

**Model**	**MAE**	**RMSE**	**WAPE**
GRU (7, imputation)	74.00	92.50	7.48%
BiLSTM (14, imputation)	97.88	118.67	9.90%
Naive	107.20	131.42	10.84%
SVR (14, null)	146.51	174.79	14.82%

#### Summary

In summary, as also observed in [27, 42], neural networks generally showed the most accurate performance among all the considered models, probably due to their ability to keep “memory” of the past within their internal representation. Of course, no one-size-fits-all solution perfectly applied to all cases. The predictions at the regional level were, in general, less accurate than those obtained on the national data. This situation was even more evident on the remaining regions. This may be due to the fact that each region has its specificity, and thus similar restriction or mobility trends did not influence the epidemiological trends in the same way for all the regions. This phenomenon has already been observed in [28], where not all US county data produced accurate predictions. This variability may have been mitigated on the national dataset due to the smoothing introduced by aggregated, higher-level features.

### Results of the what-if analysis

As already mentioned, we carried out several what-if analyses only for those scenarios in which the models exhibited the most accurate results, i.e., the national and regional datasets related to Puglia, Toscana, and Lazio. In this type of study, there is no ground truth for the alternative simulated scenarios, making it impossible to assess each scenario’s changes accurately. Therefore, in the following, we draw some hypothetical explanations. In particular, we show the percentage variation of the new positive cases predicted in each alternative scenario compared to the real underlying trend. Furthermore, since there are 20 days in the test set, we show the variations for the first ten days and the last ten days separately, to highlight whether the trend variation is somehow constant. Finally, we focus on the results in which a variation of at least 5% was observed.

#### National data

**Fig. 14:**
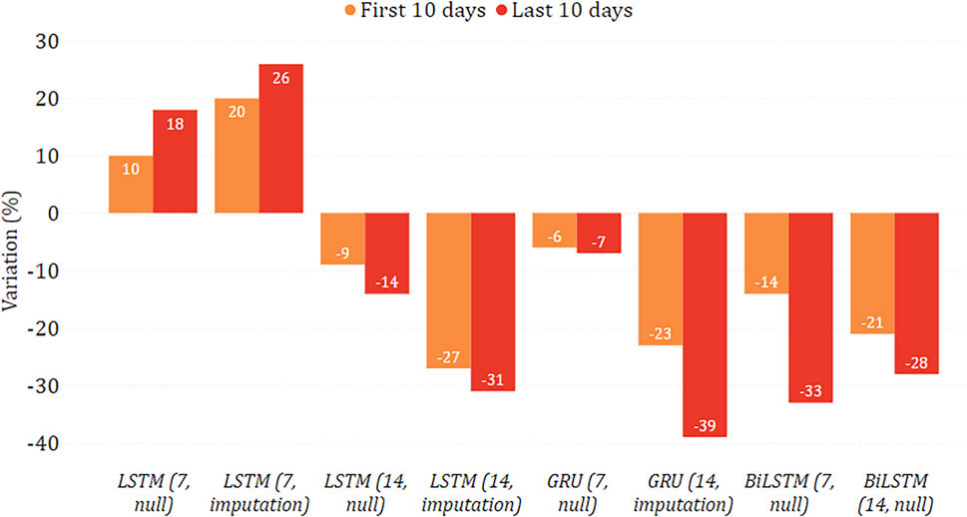
Percentage variation of new positive cases in the lockdown scenario on the national data

**Fig. 15:**
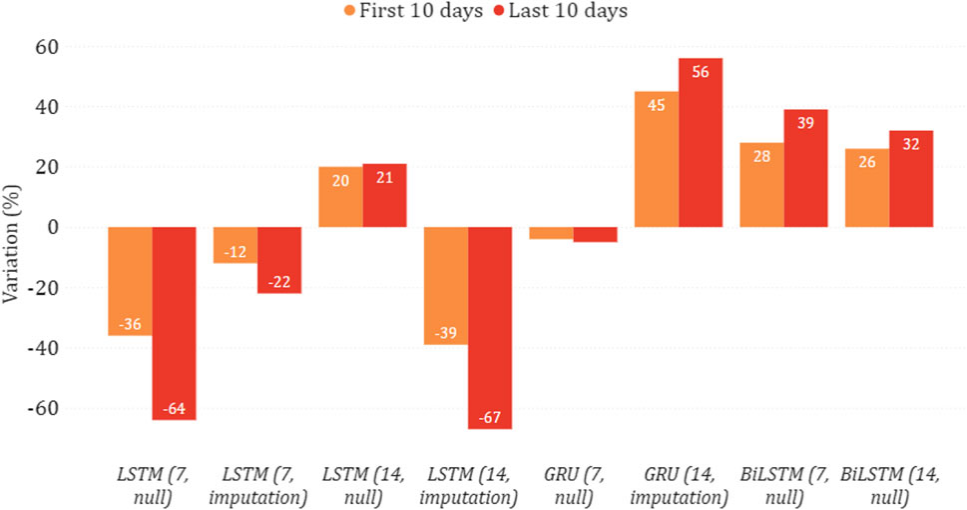
Percentage variation of new positive cases in the reopening scenario on the national data

In Figs. 14 and 15, we show the epidemiological trend predicted in the simulated scenario of total lockdown and reopening with respect to the real one. As for the former, we can observe that most models predicted a decrease in new positive cases, sometimes greater than $30\%$. As for the latter, the agreement among the models is less clear, with some counter-intuitively predicting a lower virus spread while imposing fewer restrictions. This disagreement may indicate that, unlike a lockdown, less severe restrictions do not necessarily imply a change in the trend. This may also be motivated by the fact that the testing period was already characterized by some gradual reopening initiatives, mainly due to the beginning of summer. We can also observe more evident variations between the two considered periods of ten days. This indicates that the effects of restrictive measures can be better appreciated in the long term, confirming what has already been highlighted in [28].

#### Puglia

**Fig. 16:**
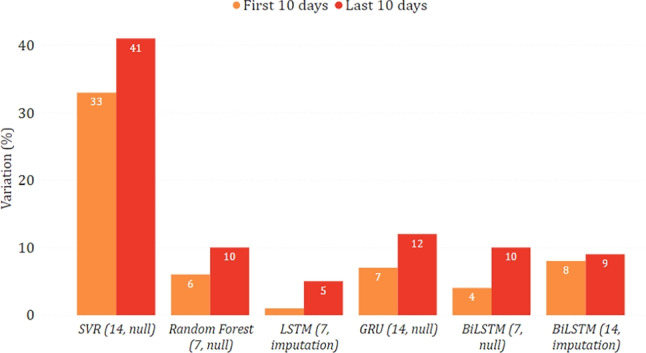
Percentage variation of new positive cases in the lockdown scenario in the case of Puglia

**Fig. 17:**
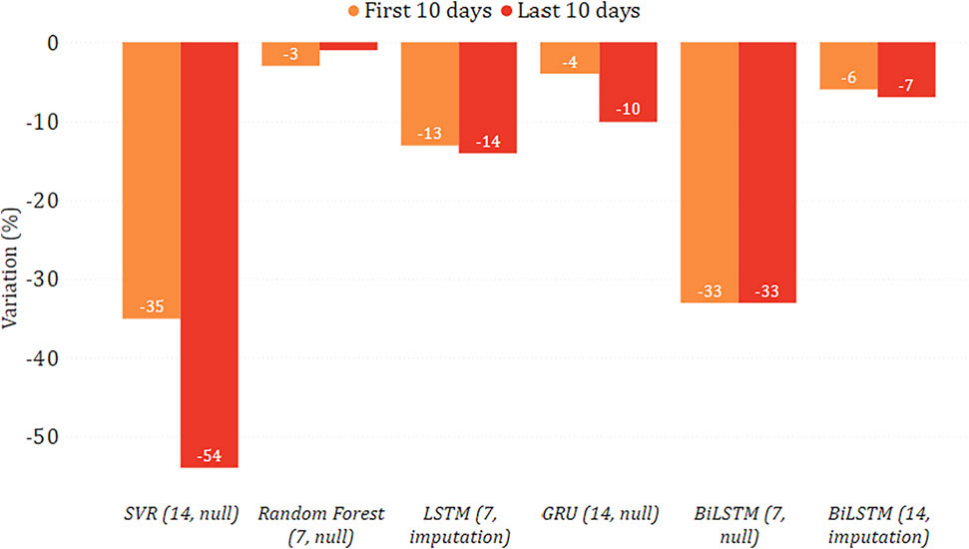
Percentage variation of new positive cases in the reopening scenario in the case of Puglia

In Figs. 16 and 17, we show the changing trend of new positive cases when simulating a lockdown or a reopening on the data related to the Puglia region. In this case, all models agree in both scenarios and predict an increase in new positive cases in the lockdown scenario and a decrease in reopening. These results could appear counter-intuitive since they indicate that a severe lockdown may make the situation worse than reopening. However, this situation becomes reasonable if we consider that severe restrictions were adopted in this region only in a much more severe epidemiological situation (first wave), which may not reflect the trends observed by the model in the testing period.

#### Toscana

**Fig. 18:**
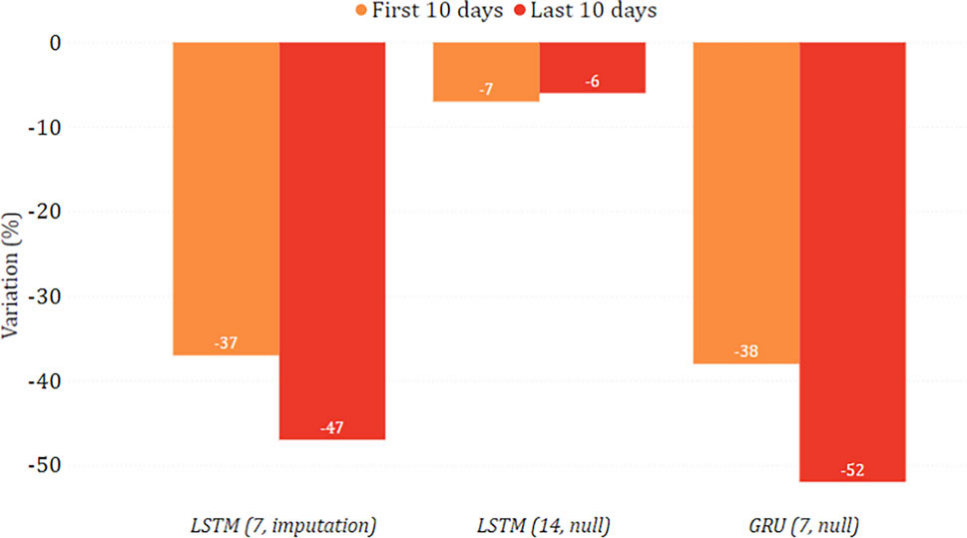
Percentage variation of new positive cases in the lockdown scenario in the case of Toscana

**Fig. 19:**
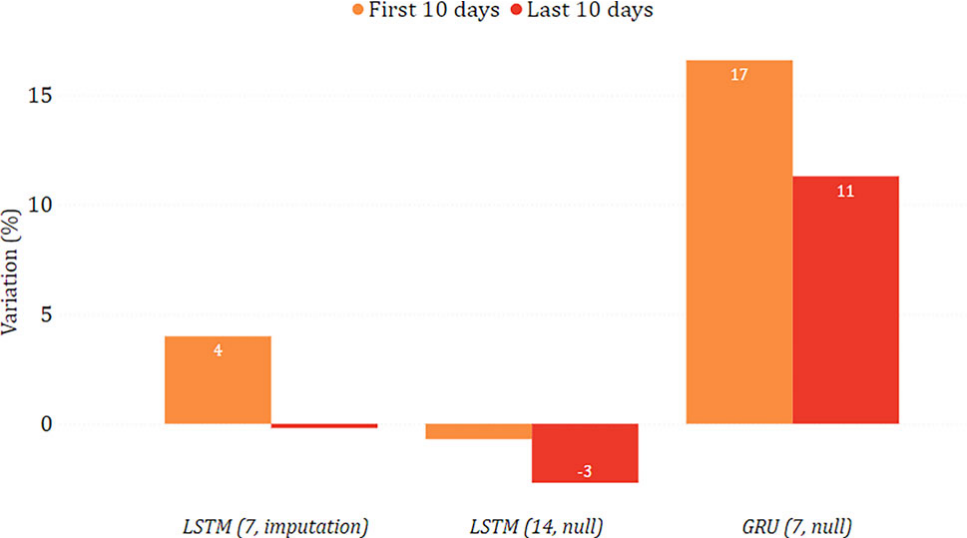
Percentage variation of new positive cases in the reopening scenario in the case of Toscana

Figures 18 and 19 show the predictions for the Toscana region. While, simulating a lockdown, the models agree that there is a reduction in new positive cases, there is less agreement in the reopening simulation, and GRU strongly suggests that the contagion would spread again. However, it can be seen that all models agree that there is a decrease after the first ten days, albeit to a different extent. This may suggest that even with a reopening, the situation can improve and possibly stabilize in the long term.

#### Lazio

**Fig. 20:**
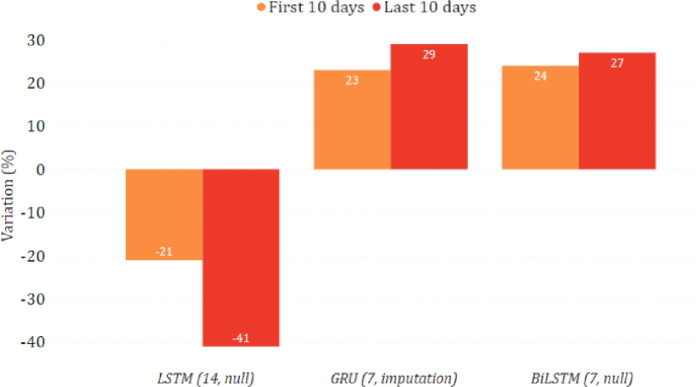
Percentage variation of new positive cases in the lockdown scenario in the case of Lazio

**Fig. 21:**
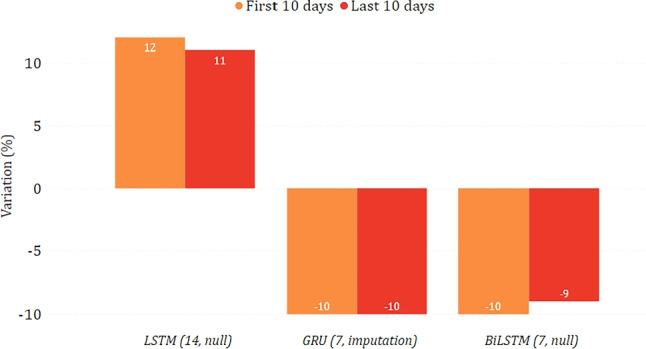
Percentage variation of new positive cases in the reopening scenario in the case of Lazio

Figures 20 and 21 show the results of the what-if analysis carried out on the Lazio region. Similar considerations with respect to those already made for the Puglia region can be drawn, as 2 out of 3 models predict an increase of new positive cases during the lockdown and a decrease after the reopening. Moreover, while a lockdown leads to a gradual change over time, reopening leads to relatively constant changes.

#### Summary

Although what-if analyses are inherently uncertain, some hypotheses can still be drawn. The first observation is that the models tend to change their predictions when a different scenario is simulated. This phenomenon confirms that mobility and restrictions affect the epidemiological trend. The disagreement between the models and some counter-intuitive results could instead be a symptom of contradictory patterns in the training data. This may also be due to the imposition of too severe restrictions too early or reopening when the situation still needed to be sufficiently safe. This seems to confirm that lockdowns as all-in-one solutions may not be effective and that applying targeted initiatives based on constant monitoring may lead to better effects on the pandemic. After the first wave, in fact, the Italian government itself began to introduce diversified policies according to individual cases. Finally, even without considering the large number of external variables we have not considered in this study, it should be noted that the behavior of the population has evolved to face the pandemic situation, and has begun to adapt to new emergencies. For example, new security protocols have been introduced, and smart working has started to spread over companies and institutions. Hence, the effectiveness of predictive models may have been influenced by concept drift phenomena introduced by these evolutions, which call for further studies on the whole historical data about COVID-19.

## Conclusion

In this paper, we applied multivariate time series forecasting methods to new positive COVID-19 cases, specifically targeting the Italian case during the first three waves. To this end, epidemiological data and data related to mobility and restrictions have been exploited to learn predictive models. This activity has been performed assuming that these features are interrelated and, therefore, should be studied jointly to obtain more reliable predictive models. Decision-makers can exploit these models to better plan intervention strategies. In addition, we performed what-if analyses to study the impact that more or less severe restrictions could have had on the spread of the virus. The results obtained seem to confirm the hypothesis that strong initiatives, like total lockdowns or total reopening, may generally not be adequate and that more specific, focused solutions should be adopted, such as those applied at the level of individual regions.

The present study outlines several points for possible further studies. First, we have limited ourselves to epidemiological, mobility, and restriction data which are unlikely to be the only relevant factors contributing to the spread of the virus: there are many other social, economic, and environmental variables that we have not considered and which may be crucial for the task at hand. Second, the temporal extension considered in this paper included only the first three waves of contagion in Italy before the new decline during the summer of 2021. Therefore, we did not study the medium-term effects of the mass vaccination campaign nor the diffusion of the several variants we have seen. However, these open up other research questions, such as the impact of adverse reactions to COVID vaccines or their effectiveness in slowing the epidemic curve, which were not the main focus of our study. Finally, from a purely methodological perspective, we point out that the smoothing effect of the data at national granularity on the fluctuations of the individual regions can be exploited by relying on *transfer learning* approaches [33, 37]. In future work, the knowledge learned about a country could be transferred to more specific data to develop more robust local predictive models.

## Data Availability

The code used to run the experiments and the complete set of results obtained are publicly available at https://doi.org/10.6084/m9.figshare.c.6299382.v1.

## Footnotes

1 https://covid19.who.int/

2 https://www.cdc.gov/coronavirus/2019-ncov/symptoms-testing/symptoms.html

3 https://www.cdc.gov/coronavirus/2019-ncov/prevent-getting-sick/how-covid-spreads.html

4 https://www.who.int/news-room/q-a-detail/coronavirus-disease-(covid-19)-vaccines

5 https://github.com/pcm-dpc/COVID-19

6 https://github.com/owid/covid-19-data

7 https://www.google.com/covid19/mobility/

8 https://covid19.apple.com/mobility
